# Surface scattering mechanisms of tantalum nitride thin film resistor

**DOI:** 10.1186/1556-276X-9-177

**Published:** 2014-04-11

**Authors:** Huey-Ru Chen, Ying-Chung Chen, Ting-Chang Chang, Kuan-Chang Chang, Tsung-Ming Tsai, Tian-Jian Chu, Chih-Cheng Shih, Nai-Chuan Chuang, Kao-Yuan Wang

**Affiliations:** 1 Department of Electrical Engineering, National Sun Yat-Sen University, Kaohsiung 804, Taiwan; 2 Department of Physics, National Sun Yat-Sen University, Kaohsiung 804, Taiwan; 3 Department of Materials and Optoelectronic Science, National Sun Yat-Sen University, Kaohsiung 804, Taiwan; 4 R&D Department, Walsin Technology Co, Kaohsiung 806, Taiwan

**Keywords:** TaN, Thin film resistor, Temperature coefficient of resistance, Surface scattering

## Abstract

In this letter, we utilize an electrical analysis method to develop a TaN thin film resistor with a stricter spec and near-zero temperature coefficient of resistance (TCR) for car-used electronic applications. Simultaneously, we also propose a physical mechanism mode to explain the origin of near-zero TCR for the TaN thin film resistor (TFR). Through current fitting, the carrier conduction mechanism of the TaN TFR changes from hopping to surface scattering and finally to ohmic conduction for different TaN TFRs with different TaN microstructures. Experimental data of current–voltage measurement under successive increasing temperature confirm the conduction mechanism transition. A model of TaN grain boundary isolation ability is eventually proposed to influence the carrier transport in the TaN thin film resistor, which causes different current conduction mechanisms.

## Background

With portable electronic devices being popular worldwide, the integration of memory [1-38], display [39-45], and IC circuits [46] has become important in the recent years. Especially, a high-accuracy thin film resistor (TFR) needs to make a light, thin, short, and small product with a decrease of tolerance for electronic and optical device applications. Tantalum nitride is a mechanically hard, chemically inner, and corrosion-resistant material and has good shock/heat resistance properties [47-50]. These properties make the material attractive for many industrial applications for use as TFR material in portable electronic products. A low or near-zero temperature coefficient of resistance (TCR) is also required for the purpose of high reliability in TFR. In order to make the TFR conform to the requirement of a stricter spec for car-used electronic applications, it is a big challenge to develop a material with near-zero TCR for a large temperature region.

In our research, a TaN thin film resistor chip was fabricated to do the current–voltage measurement and analysis. Different TaN films with different manufacture processes were applied so as to analyze characteristics of the TaN TFR. Conduction current fitting together with varied-temperature current–voltage measurement data was thoroughly investigated, from which current conduction mechanisms were determined. Finally, the TaN grain boundary isolation model was proposed to explain the current conduction mechanisms under different TaN thin film deposition conditions.

## Methods

**Figure 1:**
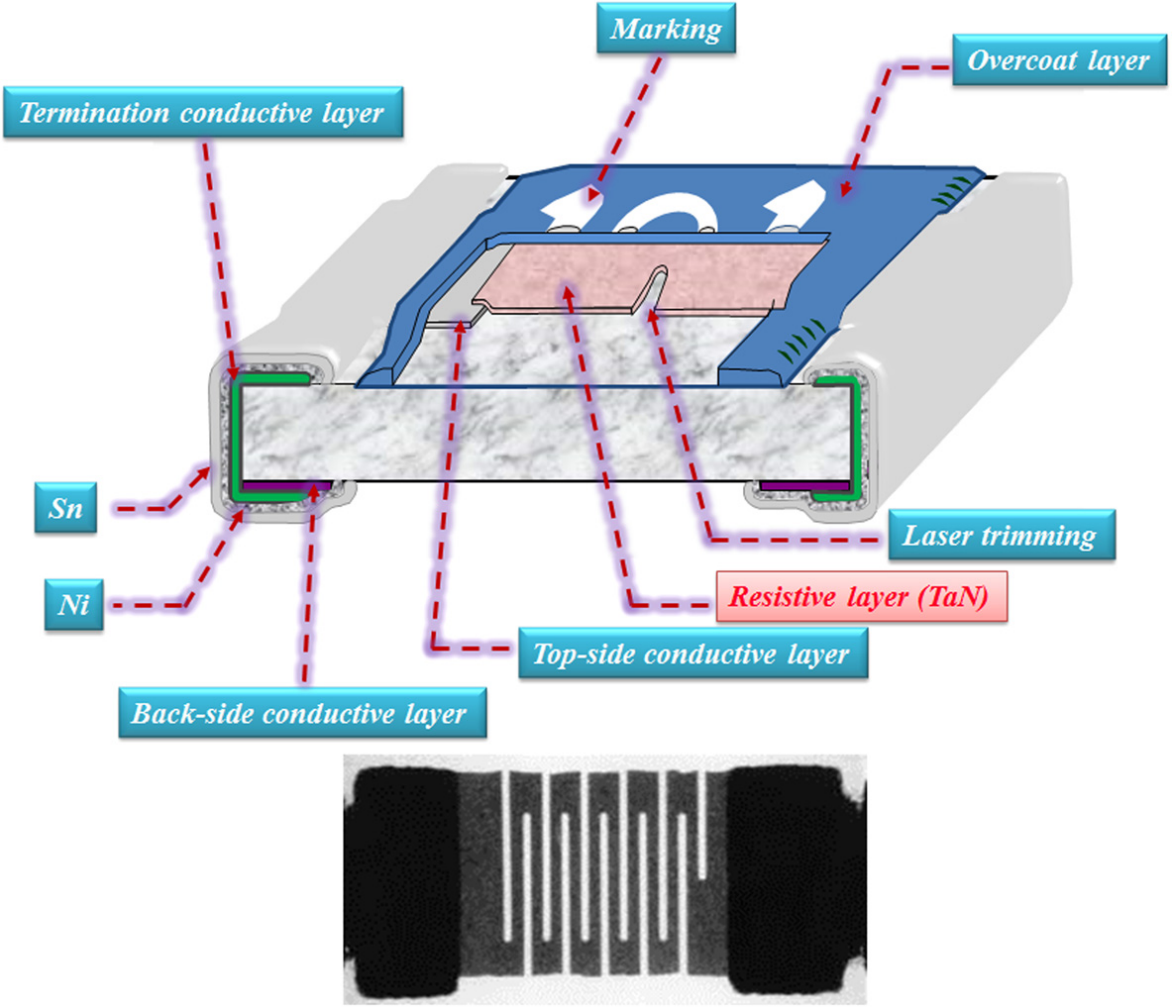
The TaN thin film resistor chip schematic structure and snake pattern photo image.

The experimental thin film resistor chips (Figure 1) were prepared as follows: Firstly, the conductor silver material was printed on an alumina substrate. Then, TaN films with a thickness of about 150 nm were deposited on the silver-printed substrate by DC sputtering with Ta target in the Ar/N_2_ mixed gas ambient. The sputtering power was fixed at a DC power of 500 W. After that, all specimens were annealed in an oven with a working pressure of 1e^-5^ Torr. The annealing process was set at different temperatures to form the TaN films with different TCR values. Finally, the thin film resistor chips were fabricated by capping a termination conductive layer through electroplating process. In order to conduct the electrical measurement and analysis of the TaN thin film resistor, a snake-type pattern (the photo image of Figure 1) was realized by the laser trimming process using a green laser to obtain higher resistance. The entire electrical measurements of devices were performed using an Agilent B1500 semiconductor parameter analyzer (Agilent Technologies Inc., Santa Clara, CA, USA).

## Results and discussion

**Figure 2:**
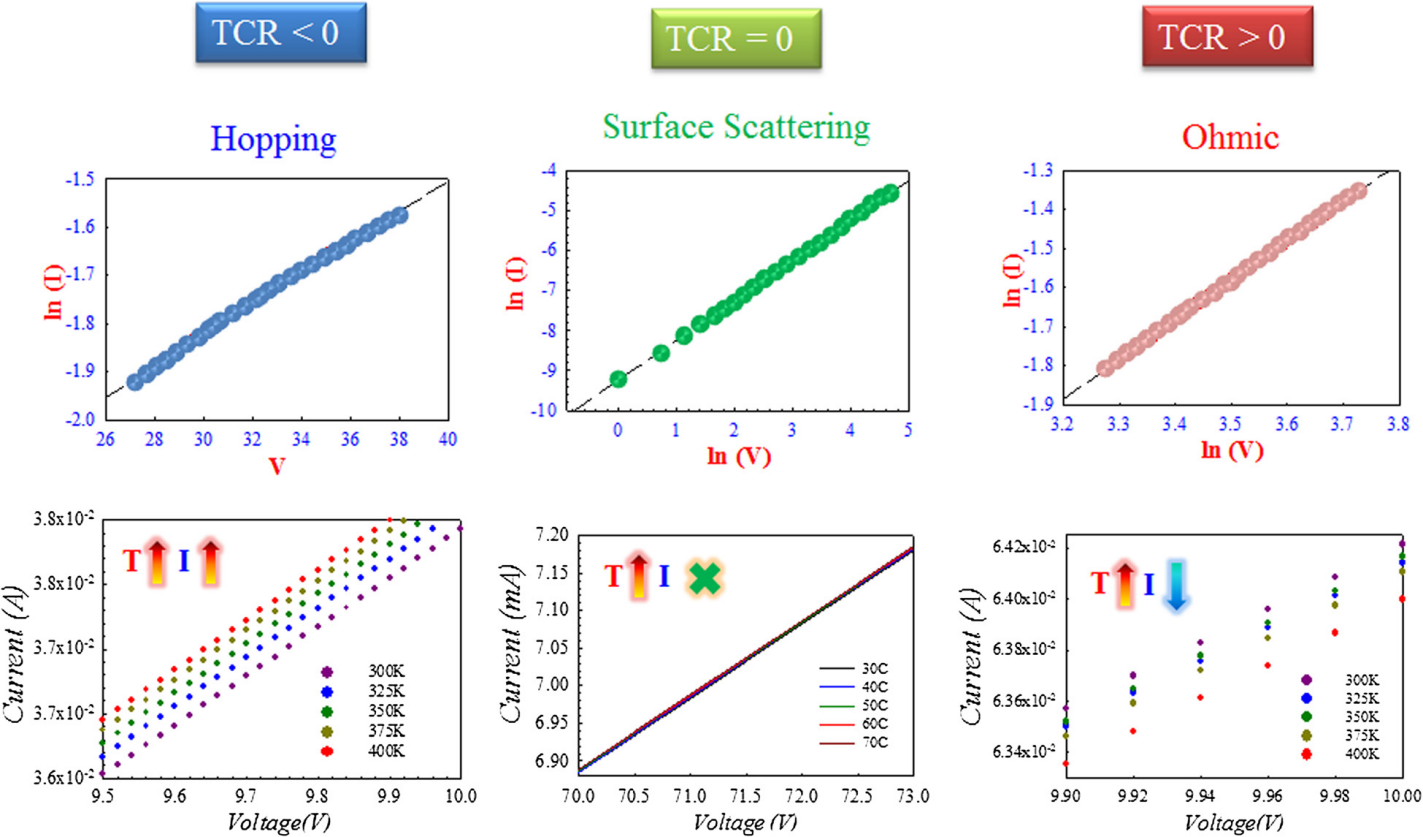
**Current conduction mechanism fitting and *I*-*V *characteristics.** Current conduction mechanism fitting and *I*-*V* characteristics of the TaN thin film resistor with different TCR values under increasing temperature environment.

DC sweeping was applied to investigate the properties of current–voltage of the TaN thin film resistor. In our experiment, we mainly focused on the current conduction mechanism in the TaN resistive layer. In order to analyze its characteristics, different TaN resistive layers with different polarities of the TCR value were employed: different TaN layers including TCR larger than zero, TCR smaller than zero, and TCR equal to zero. Through conduction current fitting, a noticeable transition of carrier conduction mechanism was found, which gradually changed from hopping conduction to surface scattering and finally to ohmic conduction with the increase of the TCR value of the TaN TFR shown in Figure 2.

To testify the validity of fitting, varied-temperature *I*-*V* measurement was applied and the results are shown in Figure 2. The TCR values in Figure 2 were <0, =0, and >0, respectively. It can be observed from the experimental data that the current of TCR < 0 was directly proportional to temperature, while the current of TCR > 0 was the opposite. The current of TCR = 0 was independent of temperature. All the experimental data were in accordance with their corresponding conduction mechanism.

**Figure 3:**
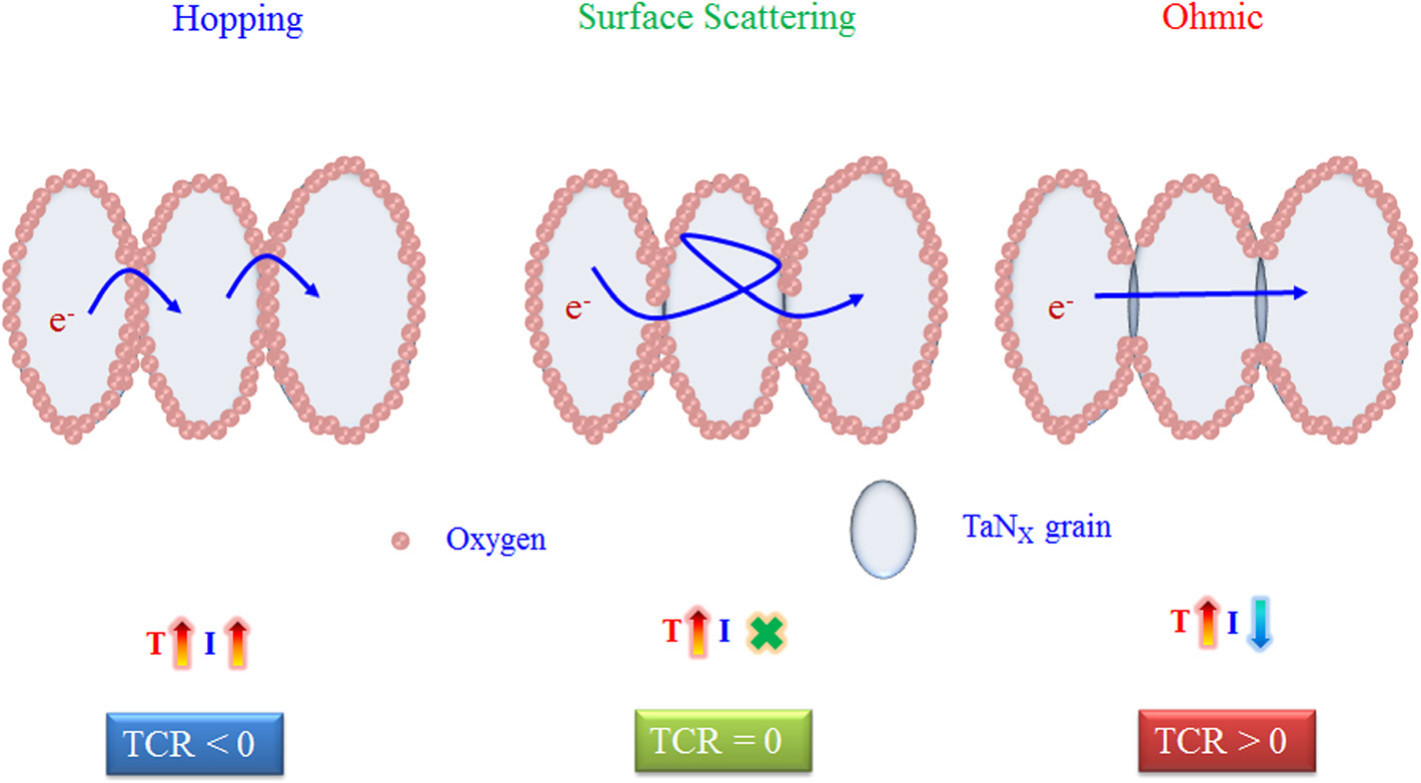
**Merging of TaN grains.** Gradual merging of TaN grains accompanied with transition of conduction mechanism for different TaN resistive layers with different TCR values.

From the experimental results, a carrier conduction model of the TaN resistive layer was proposed (Figure 3). As the oxidation of TaN grain boundary was the main reason for the change of carrier transport mechanism in the TaN resistive layer, it is easier to increase the TCR value with the increase of TaN oxidation degree. Thus, the level of oxidation in the grain boundary of TaN should be controlled carefully to achieve the target of TCR = 0. A TaN resistive layer with TCR < 0 was greatly oxidized so that the TaN grain was isolated completely, which resulted in carrier hopping conduction owing to the discrete TaN precipitates (left-side diagram of Figure 3). With the decreasing oxidation of the TaN grain boundary, the carrier conduction will be limited between two TaN grain boundaries and the surface scattering became easier for those discrete TaN grains to join and merge with each other, from which relative complete filaments can be formed, as shown in the middle diagram of Figure 3. Because of the formation of a smoother carrier conduction path and the independence of temperature, the carrier conduction mechanism transformed from hopping conduction to surface scattering (Figure 2). However, the filament is not thick enough for numerous carriers to conduct through it, which leads to the crowding of carriers. The carriers have to be forced out from the restricted filament which is also the reason why we can find space scattering conduction [51]. Meanwhile, the measurement result of Figure 2 also complies with surface scattering mechanism as current is independent with temperature. If the level of oxidation in the TaN film was decreased, ohmic conduction mechanism will dominate due to the formation of a thicker and more continuous filament (right-side diagram of Figure 3). The fitting result of ohmic conduction is shown in Figure 2.

## Conclusion

In conclusion, the carrier conduction mechanisms of TaN thin film resistors with different TCR values are thoroughly investigated. With the increase of the TCR value, the conduction mechanism transforms from hopping conduction to surface scattering and finally to ohmic conduction. The transition of the carrier conduction mechanism is explained by our model, from which the relationship of the TCR value and oxidation degree of the TaN thin film resistor can be better understood. Based on the relationship, the near-zero TCR TaN resistive layer can be fabricated by controlling the level of oxidation and can be demonstrated by electrical current measurement and fitting analysis.

## Competing interests

The authors declare that they have no competing interests.

## Authors’ contributions

HRC designed and set up the experimental procedure. YCC and TCC planned the experiments and agreed with the paper's publication. TMT revised the manuscript critically. KCC, TJC, and CCS conducted the electrical measurement of the devices. NCC fabricated the devices with the assistance of KYW. All authors read and approved the final manuscript.
